# Synthesis and Evaluation of Coumarin-Chalcone Derivatives as α-Glucosidase Inhibitors

**DOI:** 10.3389/fchem.2022.926543

**Published:** 2022-06-27

**Authors:** Chun-Mei Hu, Yong-Xin Luo, Wen-Jing Wang, Jian-Ping Li, Meng-Yue Li, Yu-Fei Zhang, Di Xiao, Li Lu, Zhuang Xiong, Na Feng, Chen Li

**Affiliations:** School of Biotechnology and Health Sciences, Wuyi University, Jiangmen, China

**Keywords:** coumarin, chalcone, α-glucosidase, enzyme inhibitor, docking

## Abstract

Coumarin and chalcone, two important kinds of natural product skeletons, both exhibit α-glucosidase inhibitory activity. In this work, coumarin-chalcone derivatives 3 (**a∼v**) were synthesized, and their α-glucosidase inhibitory activity was screened. The results showed that all synthetic derivatives (IC_50_: 24.09 ± 2.36 to 125.26 ± 1.18 μM) presented better *α*-glucosidase inhibitory activity than the parent compounds 3-acetylcoumarin (IC_50_: 1.5 × 10^5^ μM) and the positive control acarbose (IC_50_: 259.90 ± 1.06 μM). Among them, compound **3t** displayed the highest *α*-glucosidase inhibitory activity (IC_50_: 24.09 ± 2.36 μM), which was approximately 10 times stronger than that of acarbose. The kinetic assay of **3t** (*K*_I_ = 18.82 μM, *K*_IS_ = 59.99 μM) revealed that these compounds inhibited α-glucosidase in a mixed-type manner. Molecular docking was used to simulate the interaction between α-glucosidase and compound **3t**.

## Introduction

Type 2 diabetes mellitus (T2DM) is a metabolic disease characterized by hyperglycemia resulting from insulin resistance and insufficient insulin secretion by pancreatic β-cells. One of the key reasons for the hyperglycemia is the enzymatic hydrolysis of carbohydrates. α-Glucosidase (EC 3.2.1.20) plays an important role in carbohydrate digestion, in which the oligosaccharides and disaccharides from dietary carbohydrates are broken down into monosaccharides. The α-glucosidase inhibitors suppress the absorption and assimilation of monosaccharides and delay the digestion of carbohydrates (Cohen and Goedert, 2004; Proença et al., 2017; Xu et al., 2019; Zhong et al., 2019). Some commercially available α-glucosidase inhibitors, including miglitol, voglibose, and acarbose, have been used in the clinical treatment of T2DM, but they still show several adverse effects (Chai et al., 2015; Khursheed et al., 2019; Rocha et al., 2019). In addition, α-glucosidase is closely related to hepatitis, cancer, and Pompe disease (Kasturi et al., 2018; Gulcin et al., 2019). Therefore, it is always beneficial in medicinal chemistry to develop potent α-glucosidase inhibitors.

**FIGURE 1:**
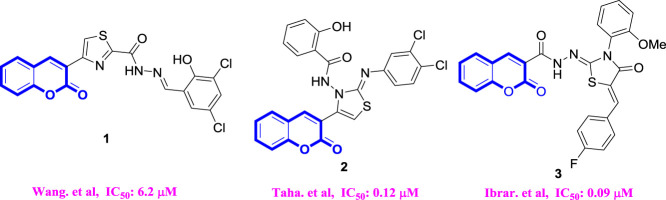
α-glucosidase inhibitors containing coumarin.

Coumarin is an important natural product skeleton with various pharmacological properties; among these, its anti-hyperglycemic activity is the focus of our research (Kontogiorgis et al., 2012; Katsori and Litina, 2014; Adib et al., 2018). Previous studies have shown that natural products containing the coumarin moiety and synthesized coumarin derivatives exhibit anti-hyperglycemic activity through the inhibition of α-glucosidase (Adib et al., 2018). For instance, Wang et al. (2016) reported on a series of coumarin-thiazoles with the highest α-glucosidase inhibitory activity (IC_50_ = 6.2 μM). Salar et al. (2016) developed 3-thiazolyl coumarins with the most potent α-glucosidase inhibitory activity (IC_50_ = 0.12 μM) (Salar et al., 2016)). Ibrar *et al.* designed coumarinyl iminothiazolidinones with the most effective inhibitory activity (IC_50_ = 0.09 μM) (Ibrar et al., 2017). (Figure 1) Chalcone, an important sub-structure widely existing in many natural products, has the ability to bind to a variety of targets, resulting in many biological activities (Bak et al., 2011; Feng et al., 2014; Kang et al., 2018; Djemoui et al., 2020; Rocha et al., 2020; Dorn et al., 2010).

In medicinal chemistry, the hybrid of pharmacophore and skeleton is an effective strategy for obtaining active lead compounds. Until now, many coumarin-chalcone derivatives had been synthesized with many biological properties, such as antioxidant, anti-cancer, antibacterial, and anti-inflammatory properties (Pingaew et al., 2014; Seidel et al., 2014; Lee et al., 2018). However, there were few reports on the application of α-glucosidase inhibitors. Therefore, we synthesized coumarin-chalcone derivatives **3a∼v** and screened their inhibitory activity against α-glucosidase.

## Results and Discussion

### Chemistry

**SCHEME 1:**
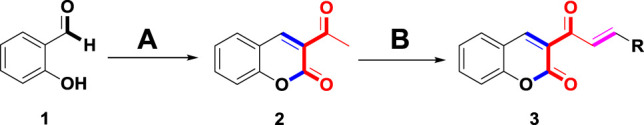
The synthetic route to coumarin-chalcone derivatives 3 (a∼v). Reagents and conditions: **(A)** ethyl acetoacetate, piperidine, ethanol, 65°C, 20 min **(B)** substituted aldehydes, piperidine, ethanol, reflux, 24 h.

Coumarin-chalcone derivatives **3(a∼v)** were prepared according to a well-known method (Roussel and Fraser, 1993; Vazquez-Rodriguez et al., 2015; Shang et al., 2018; Wang et al., 2019). In the presence of piperidine, salicylaldehyde **1**) reacted with ethyl acetoacetate to produce 3-acetylcoumarin (**2**). Then 3-acetylcoumarin **2**) and the substituted aldehydes underwent the aldol condensation reaction under the catalysis of piperidine to give coumarin-chalcone derivatives **3(a∼v)** (Scheme 1). Compounds **3(a∼v)** had been reported previously and the title compounds were characterized by ^1^H NMR.

### α-Glucosidase Inhibition Assay

Coumarin-chalcone derivatives **3(a∼v)** were screened for their inhibitory activities against α-glucosidase using 4-nitrophenyl-α-D-galactopyranoside (*p*-NPG) as a substrate and the results are summarized in Table 1. The parent compounds 3-acetylcoumarin only showed low inhibitory activity with IC_50_ values of 1.5 × 10^5^. Interestingly, all synthetic derivatives showed moderate to good inhibitory activity towards α-glucosidase with IC_50_ values ranging from 24.09 ± 2.36 to 125.26 ± 1.18 µM. The results revealed that the inhibitory activities of synthetic compounds were significantly enhanced by hybridizing the two molecular skeletons. Furthermore, all the title compounds presented higher inhibitory activity than that of the positive control acarbose (IC_50_: 259.90 ± 1.06 μM). Among them, compounds **3j**, **3q** and **3t** demonstrated the highest inhibitory activity (IC_50_: 30.30 ± 2.53, 29.74 ± 2.68, and 24.09 ± 2.36 μM, respectively): 10 times stronger than that of acarbose.

**TABLE 1:** α-Glucosidase inhibitory activities of compounds **3** (**a∼v**).

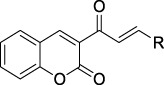					
**Compound**	**R**	**IC_50_ (μM)**	**Compound**	**R**	**IC_50_ (μM)**
3a	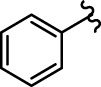	125.26 ± 1.18	3b	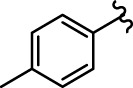	95.23 ± 1.35
3c	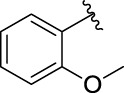	60.89 ± 2.74	3d	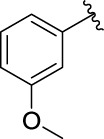	96.39 ± 1.37
3e	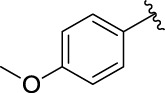	105.18 ± 1.98	3f	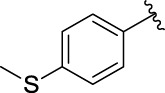	75.53 ± 0.98
3g	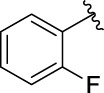	48.36 ± 1.42	3h	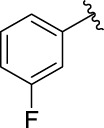	45.68 ± 1.28
3i	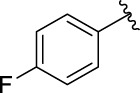	35.68 ± 0.28	3j	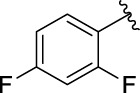	30.30 ± 2.53
3k	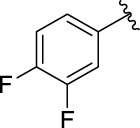	49.68 ± 3.28	3l	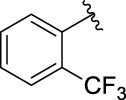	71.52 ± 2.14
3m	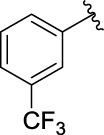	64.71 ± 1.82	3n	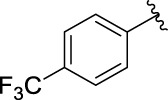	53.58 ± 1.95
3o	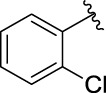	59.68 ± 1.73	3p	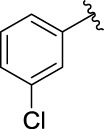	52.62 ± 2.45
3q	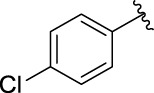	29.74 ± 2.68	3r	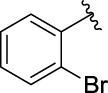	38.56 ± 1.87
3s	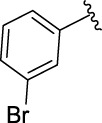	35.56 ± 2.18	3t	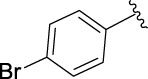	24.09 ± 2.36
3u	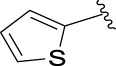	109.23 ± 2.69	3v	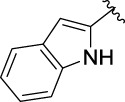	103.31 ± 1.45
3-Acetylcoumarin		1.5 × 105			
Acarbose		259.90 ± 1.06			

### Structure Activity Relationships

The structure activity relationships (SARs) of compounds **3(a∼v)** was analyzed based on their α-glucosidase inhibitory activities. Compound **3a** (IC_50_: 125.26 ± 1.18 μM) without any substituent was selected as the template compound. It could be seen that the introduction of various substituents resulted in an obvious change in inhibitory activity. For compound **3b** (IC_50_: 95.23 ± 1.35 μM) with a 4-methyl group, its inhibitory activity slightly increased compared to **3a**. For compounds **3(g∼i)** with the fluorine group, **3(l∼n)** with the trifluoromethyl group, **3(o∼q)** with the chlorine group, and **3(r∼t)** with the bromine group, all presented stronger inhibitory activity than compound **3a**, indicating that electron-withdrawing groups such as fluorine, trifluoromethyl, chlorine, and bromine could lead to an increase in inhibitory activity. Among them, **3i** with the 4-fluorine group (IC_50_: 35.68 ± 0.28 μM), **3n** with the 4-trifluoromethyl group (IC_50_: 53.58 ± 1.95 μM), **3q** with the 4-chlorine group (IC_50_: 29.74 ± 2.68 μM), and **3t** with the 4-bromine group (IC_50_: 24.09 ± 2.36 μM) showed higher inhibitory activity than the 2- and 3-position groups. For compounds **3j** and **3k** with difluoro groups, the introduction of 2,4-difluoro groups (**3j**, IC_50_: 30.30 ± 2.53) resulted in the stronger inhibitory activities. While for compounds **3(c∼e)** with methoxy group, the 2-position group (**3c**, IC_50_: 60.89 ± 2.74) was better than 3-position group and 4-position group.

Furthermore, the sequencing of inhibitory activity was identified: **3t** (with 4-bromine group) > **3q** (with 4-chlorine group) > **3i** (with 4-fluorine group) > **3n** (with 4-trifluoromethyl group), predicting that stronger electron-withdrawing groups led to weaker inhibitory activity as for the compounds with electron-withdrawing groups. In addition, in the electron-withdrawing groups, inhibitory activity was related to the substituted position as follows: the inhibitory activity of compounds with the withdrawing groups at para-position was superior to that at meta-position, which is better than that at ortho-position. The introduction of thiophene (**3u**) or indole (**3v**) ring only slightly improved the inhibitory activity compared with compound **3a.**

### Inhibitory Mechanism Analysis

**FIGURE 2:**
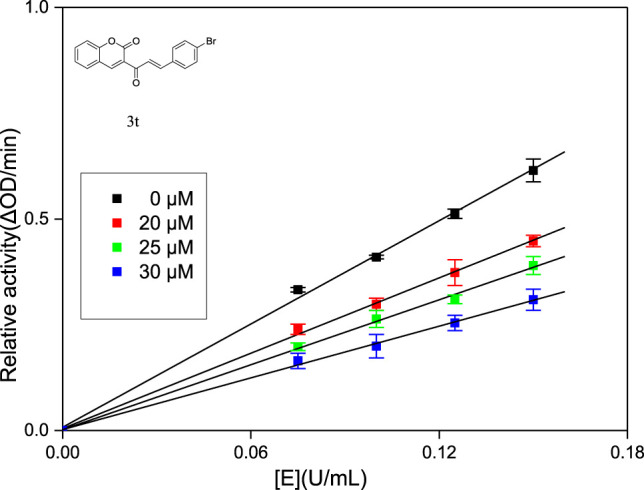
Inhibition mechanism determination of compounds **3t** on α-glucosidase.

Generally, according to the type of inhibition, enzyme inhibitors can be divided into reversible inhibitors and irreversible inhibitors (Abuelizz et al., 2019). In order to obtain the principle of the combination of enzyme inhibitors and enzymes, it is necessary to study the interaction between enzyme inhibitors and enzymes. Compounds **3j, 3q** and **3t** with strongest inhibitory activity were chosen for the research of inhibition kinetics against α-glucosidase (the inhibitory mechanism analysis of compound **3t** was shown in Figure 2 and figures for the inhibitory mechanism analysis of compounds **3j** and **3q** have been shown in the supporting information). A series of plots of enzymatic reaction rate (v) vs. α-glucosidase concentration in the presence of inhibitors were generated to identify the type of inhibition that is listed in Figure 1. The presence of **3j**, **3q** and **3t** did not change the number of enzymes but reduced the enzyme activity, which indicated that their inhibition mechanisms on α-glucosidase were reversible.

**FIGURE 3:**
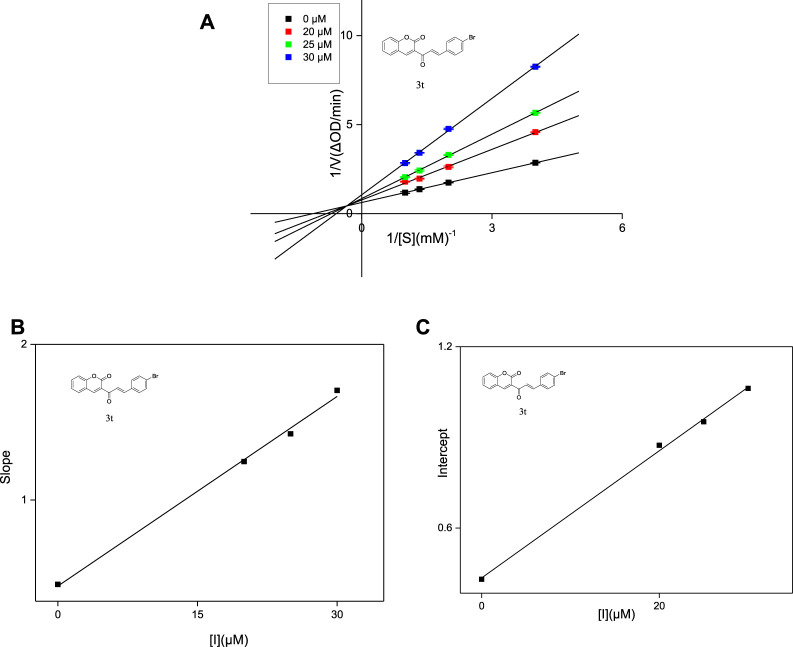
Lineweaver-Burk plots of compounds **3t** on α-glucosidase **(A)**. Plot of slope vs. the concentration of compounds for the calculation of the inhibition constant K_I_ **(B)**. Plot of intercept vs. the concentration of compounds for the determination of the inhibition constant K_IS_ **(C)**.

The inhibit type of inhibitors on α-glucosidase include four types, named competitive inhibition, non-competitive inhibition, mixed inhibition, and anti-competitive inhibition (Abuelizz et al., 2019). The inhibition modes of compounds **3j**, **3q** and **3t** against α-glucosidase were investigated using Lineweaver-Burk double reciprocal plot. As shown in Figure 3, the straight lines of 1/v vs. 1/(S) in the presence of compounds **3j**, **3q** and **3t** intersected at a point in the second quadrant respectively, illustrating that the inhibit type of **3j**, **3q** and **3t** was mixed-type inhibition. Subsequently, the K_I_ values and K_IS_ values of **3j**, **3q** and **3t** were calculated based on the slope or intercept vs. PNPG concentration and summarized in Table 2. The higher K_IS_ values compared to K_I_ values indicated that the affinity of compounds **3j**, **3q** and **3t** with free enzyme was higher than that with enzyme-substrate complex.

**TABLE 2:** Type of inhibition mechanism, as well as K_I_ and K_IS_ values of compounds **3j**, **3q** and **3t**.

Compound	Inhibition mechanism	K_I_ (μM)	K_IS_ (μM)
3j	Mixed type	19.53	25.94
3q	Mixed type	16.13	20.34
3t	Mixed type	11.02	20.71

### Molecular Docking Simulation

**FIGURE 4:**
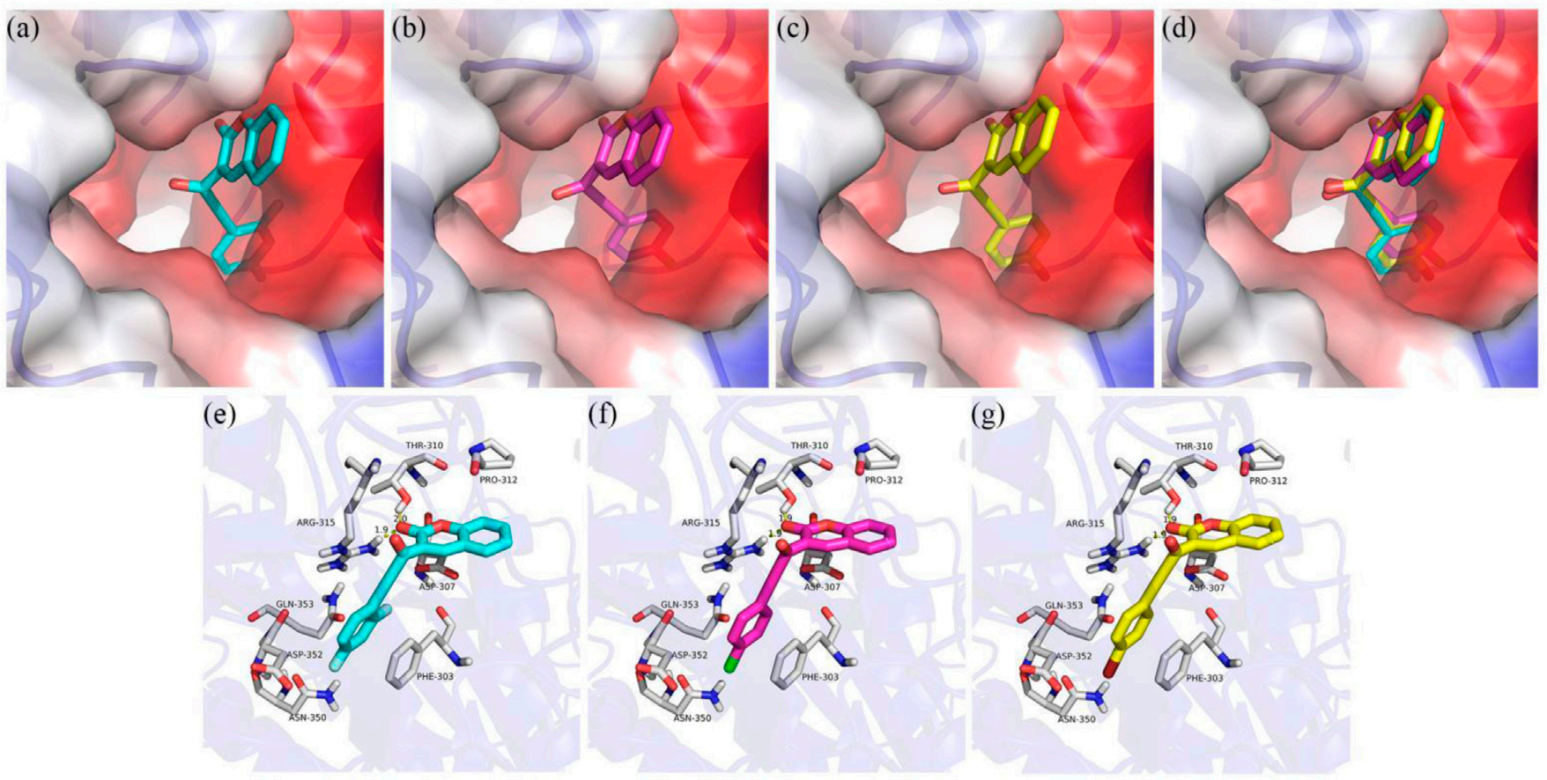
Molecular docking of compounds **3d**, **3f** and **3i** with α-glucosidase. **(A)** Compound **3q** in the active pocket **(B)**; Compound **3t** in the active pocket **(C)**; compounds **3j**, **3q** and **3t** in the active pocket of α-glucosidase **(D)**; 2D view of **3j** with α-glucosidase **(E)**; 2D view of **3q** with α-glucosidase **(F)**; 2D view of **3t** with α-glucosidase **(G)**.

To better understand the inhibition mechanism of compounds **3j**, **3q** and **3t**, the binding modes of α-glucosidase with **3j**, **3q** and **3t** were simulated using Sybyl 2.1.1 (United States) and Pymol software. The crystal structure of *Saccharomyces cerevisiae* isomaltase (PDB: 3AJ7) with 72.4% of sequence identity with α-glucosidase was chosen as the target protein (Wang et al., 2017; Asgari et al., 2019; Morocho et al., 2019; Salar et al., 2016). As can be seen in Figures 4A–D, compounds **3j**, **3q** and **3t** had the similar interaction with the active pocket of α-glucosidase.. Figures 4E–G show that the carbonyl group of coumarin of **3j**, **3q** and **3t** all formed two hydrogen bonds with Thr310 and Arg315, respectively. Compounds **3j**, **3q** and **3t** all made an π-π interaction with Phe303; and all established hydrophobic interactions with Pro310, Asp307, Asp352, Gln353, and Asn350.

## Conclusion

In summary, the α-glucosidase inhibitory activity of coumarin-chalcone derivatives **3(a∼v)** was evaluated. The results showed that all compounds presented outstanding α-glucosidase inhibitory activities (IC_50_: 24.09 ± 2.36 to 125.26 ± 1.18 μM) than the positive control acarbose and parent compounds 3-acetylcoumarin and benzaldehyde. Compounds **3j, 3q, 3t** displayed the highest α-glucosidase inhibitory activity (IC_50_: 30.30 ± 2.53, 29.74 ± 2.68, 24.09 ± 2.36μM, respectively), which was approximately 10 times stronger than acarbose. Inhibition mechanism results revealed that these compounds inhibited α-glucosidase in a mixed-type manner. Molecular docking verified the interactions of α-glucosidase with compounds **3j**, **3q**, and **3t**.

## Experiment

### Chemicals and Instruments

Ethyl acetoacetate, salicylaldehyde and absolute ethanol were analytical pure grade and purchased from Aladdin (Shanghai) Reagent Co., Ltd. Piperidine; glacial acetic acid, petroleum ether and ethyl acetate were supplied by Titan (Shanghai) Technology Co., Ltd.; α-Glucosidase from *Saccharomyces cerevisiae* (EC 3.2.1.20), 4-nitrophenyl-α-D-galactopyranoside (*p*-NPG), and Dimethyl sulfoxide (DMSO) were supported by Sigma-Aldrich (United States) Chemical Co., Ltd. Melting points were tested on a micro melting-point instrument. ^1^H NMR spectra were measured (CDCl_3_) by Bruker DPX-500 MHz AVANCE with TMS as an internal standard. Mass spectroscopy was performed on a (LCQTM). The absorbance was recorded by a micro-plate reader.

### Synthesis of 3-Acetylcoumarin

To a solution of Salicylaldehyde **1** (1.0 mmol) in ethanol (10 ml), ethyl acetoacetate (1.0 mmol) and piperidine (1.0 mmol) were added and the mixture was stirred at 65°C for 20 min. When the reaction was judged to be complete by TLC, the crude product was obtained by filtration, followed by washing with petroleum ether to produce 3-acetylcoumarin **2**. Yellow solid; yield 72.3%; ^1^H NMR (500 MHz, Chloroform-*d*) *δ* 8.54 (s, ^1^H), 7.70–7.66 (m, 2H), 7.42–7.36 (m, 2H) 2.76 (s, 3H).

### Synthesis of Coumarin-Chalcone Derivatives 3(a∼v)

To a solution of 3-acetylcoumarin **2** (1.0 mmol) in ethanol (10 ml) substituted aromatic aldehydes (1.0 mmol) and piperidine (1.0 mmol) were added, and then the mixture was refluxed for 24 h. The crude product was obtained by filtration, and subsequently by recrystallization by ethanol to give the title compounds **3(a∼v)**.

**(*E*)-3-cinnamoyl-2H-chromen-2-one (3a)**. Yellow solid; yield 51.7%; ^1^H NMR (500 MHz, Chloroform-*d*) *δ* 8.60 (s, ^1^H), 7.92 (dd, *J* = 40, 20 Hz, 2H), 7.71–7.65 (m, 4H), 7.44–7.39 (m, 4H), 7.35 (t, *J* = 8, 7.5 Hz, ^1^H).

**(*E*)-3-[3-(p-tolyl) acryloyl]-2H-chromen-2-one (3b)**. Yellow solid; yield 45.9%; ^1^H NMR (500 MHz, Chloroform-*d*) *δ* 8.58 (s, ^1^H), 7.84 (dd, *J* = 20, 15 Hz, 2H), 7.69–7.62 (m, 4H), 7.39 (d, *J* = 5 Hz, ^1^H), 7.35 (td, *J* = 7.5, 1 Hz, ^1^H), 6.93 (dt, *J* = 10, 3 Hz, 2H), 3.85 (s, 3H).

**(*E*)-3-[3-(2-methoxyphenyl) acryloyl]-2H-chromen-2-one (3c)**. Yellow solid; yield 37.7%; ^1^H NMR (500 MHz, Chloroform-*d*) *δ* 8.56 (s, ^1^H), 8.22 (d, *J* = 15 Hz, ^1^H), 7.98 (d, *J* = 15 Hz, ^1^H), 7.73–7.62 (m, 3H), 7.42–7.32 (m, 3H), 6.99 (t, *J* = 10, 10 Hz, ^1^H), 6.93 (d, *J* = 10 Hz, ^1^H), 3.92 (s, 3H).

**(*E*)-3-[3-(3-methoxyphenyl) acryloyl]-2H-chromen-2-one (3d)**. yellow solid; yield 42.1%; ^1^H NMR (500 MHz, Chloroform-*d*) *δ* 8.59 (s, ^1^H), 7.93 (d, *J* = 20 Hz, ^1^H), 7.71–7.65 (m, 2H), 7.41 (d, *J* = 5 Hz, ^1^H), 7.39–7.31 (m, 2H), 7.28 (d, *J* = 10 Hz, ^1^H), 7.19 (t, *J* = 5, 2 Hz, ^1^H), 6.97 (ddd, *J* = 8, 2.5, 1 Hz, ^1^H), 3.86 (s, 3H).

**(*E*)-3-[3-(4-methoxyphenyl) acryloyl]-2H-chromen-2**-one (3e). Yellow solid; Yield 49.7%; ^1^H NMR (500 MHz, Chloroform-*d*) *δ* 8.58 (s, ^1^H), 7.88 (dd, *J* = 30, 15 Hz, 2H), 7.69–7.64 (m, 2H), 7.58 (d, *J* = 10 Hz, 2H), 7.40 (d, *J* = 10 Hz, ^1^H), 7.35 (t, *J* = 10, 5 Hz, ^1^H), 7.22 (d, *J* = 10 Hz, 2H), 2.39 (s, 3H).

**(*E*)-3-{3-[4-(methylthio)phenyl**] **acryloyl}-2H-chromen-2-one (3f)**. Yellow solid; yield 55.2%; ^1^H NMR (500 MHz, Chloroform-*d*) *δ* 8.59 (s, ^1^H), 7.87 (dd, *J* = 40, 20 Hz, 2H), 7.70–7.64 (m, 2H), 7.61–7.57 (m, 2H), 7.40 (d, *J* = 10 Hz, ^1^H), 7.35 (td, *J* = 10, 1.5 Hz, ^1^H), 7.24 (d, *J* = 10 Hz, 2H), 2.52 (s, 3H).

**(*E*)-3-[3-(2-fluorophenyl) acryloyl]-2H-chromen-2-one (3g).** Yellow solid; yield 45.0%; ^1^H NMR (500 MHz, Chloroform-*d*) *δ* 8.60 (s, ^1^H), 8.01 (dd, *J* = 30, 15 Hz, 2H), 7.74 (t, *J* = 10, 10 Hz, ^1^H), 7.71–7.65 (m, 2H), 7.42–7.34 (m, 3H), 7.19 (t, *J* = 10, 10 Hz, ^1^H), 7.12 (t, *J* = 10, 10 Hz, ^1^H).

**(*E*)-3-[3-(3-fluorophenyl) acryloyl]-2H-chromen-2-one (3h)**. Yellow solid; yield 35.3%; ^1^H NMR (500 MHz, Chloroform-*d*) *δ* 8.61 (s, ^1^H), 7.95 (d, *J* = 20 Hz, ^1^H), 7.81 (d, *J* = 15 Hz, ^1^H), 7.71–7.66 (m, 2H), 7.45–7.36 (m, 5H), 7.19 (tdd, *J* = 8.2, 2.6, 1 Hz, ^1^H).

**(*E*)-3-[3-(4-fluorophenyl) acryloyl]-2H-chromen-2-one (3i)**. Yellow solid; yield 44.9%; ^1^H NMR (500 MHz, Chloroform-*d*) *δ* 8.61 (s, ^1^H), 7.90 (d, *J* = 20 Hz, ^1^H), 7.84 (d, *J* = 20 Hz, ^1^H), 7.71–7.65 (m, 4H), 7.41 (d, *J* = 10 Hz, ^1^H), 7.37 (td, *J* = 8.1, 0.4 Hz, ^1^H), 7.14–7.08 (m, 2H).

**(*E*)-3-[3-(2,4-difluorophenyl) acryloyl]-2H-chromen-2-one (3j).** Yellow solid; yield 44.7%; ^1^H NMR (500 MHz, Chloroform-*d*) *δ* 8.61 (s, ^1^H), 7.96 (d, *J* = 15 Hz, 2H), 7.78–7.71 (m, ^1^H), 7.71–7.65 (m, 2H), 7.41 (d, *J* = 10 Hz, ^1^H), 7.39–7.35 (m, ^1^H), 6.98–6.92 (m, ^1^H), 6.91–6.84 (m, ^1^H).

**(*E*)-3-[3-(3,4-difluorophenyl) acryloyl]-2H-chromen-2-one (3k).** Yellow solid; yield 37.3%; ^1^H NMR (500 MHz, Chloroform-*d*) *δ* 8.64 (s, ^1^H), 7.91 (d, *J* = 15 Hz, ^1^H), 7.78 (d, *J* = 15 Hz, ^1^H), 7.70 (dd, *J* = 10, 5 Hz, ^1^H), 7.56–7.50 (m, ^1^H), 7.45–7.38 (m, 4H), 7.23 (dt, *J* = 10, 10 Hz, ^1^H).

**(*E*)-3-{3-[2-(trifluoromethyl) phenyl]acryloyl**}**-2H-chromen-2-one (3l).** Yellow solid; yield 39.8%; ^1^H NMR (500 MHz, Chloroform-*d*) *δ* 8.63 (s, ^1^H), 7.94 (dd, *J* = 10, 5 Hz, 2H), 7.74–7.58 (m, 4H), 7.53–7.48 (m, ^1^H), 7.45–7.34 (m, 3H).

**(*E*)-3-{3-[3-(trifluoromethyl) phenyl]acryloyl**}**-2H-chromen-2-one (3m)**. Yellow solid; yield 39.9%; ^1^H NMR (500 MHz, Chloroform-*d*) *δ* 8.62 (s, ^1^H), 7.86 (dd, *J* = 10, 5 Hz, 3H), 7.71–7.65 (m, 3H), 7.57–7.53 (m, ^1^H), 7.44–7.34 (m, 3H).

**(*E*)-3-{3-[4-(trifluoromethyl) phenyl]acryloyl**}**-2H-chromen-2-one (3n).** Yellow solid; yield 41.4%; ^1^H NMR (500 MHz, Chloroform-*d*) *δ* 8.63 (s, ^1^H), 7.86 (d, *J* = 15 Hz, ^1^H), 7.78 (d, *J* = 10 Hz, 2H), 7.72–7.65 (m, 3H), 7.44–7.36 (m, 3H).

**(*E*)-3-[3-(2-chlorophenyl) acryloyl]-2H-chromen-2-one (3o).** Yellow solid; yield 51.2%; ^1^H NMR (500 MHz, Chloroform-*d*) *δ* 8.61 (s, ^1^H), 8.03 (d, *J* = 15 Hz, ^1^H), 7.79 (d, *J* = 15 Hz, ^1^H), 7.70–7.66 (m, 3H), 7.58 (d, *J* = 10 Hz, ^1^H), 7.46–7.35 (m, 4H).

**(*E*)-3-[3-(3-chlorophenyl) acryloyl]-2H-chromen-2-one (3p).** Yellow solid; yield 36.4%; ^1^H NMR (500 MHz, Chloroform-*d*) *δ* 8.61 (s, ^1^H), 7.95 (d, *J* = 15 Hz, ^1^H), 7.78 (d, *J* = 15 Hz, ^1^H), 7.71–7.64 (m, 3H), 7.55 (d, *J* = 10 Hz, ^1^H), 7.43–7.34 (m, 4H).

**(*E*)-3-[3-(4-chlorophenyl)acryloyl**]**-2H-chromen-2-one (3q).** Yellow solid; yield 45.2%; ^1^H NMR (500 MHz, Chloroform-*d*) *δ* 8.61 (s, ^1^H), 7.93 (d, *J* = 15 Hz, ^1^H), 7.81 (d, *J* = 15 Hz, ^1^H), 7.70–7.65 (m, 2H), 7.63 (dt, *J* = 8.5, 2.5 Hz, 2H), 7.42–7.34 (m, 4H).

**(*E*)-3-[3-(2-bromophenyl)acryloyl**]**-2H-chromen-2-one (3r).** Yellow solid; yield 40.8%; ^1^H NMR (500 MHz, Chloroform-*d*) *δ* 8.62 (s, ^1^H), 8.24 (d, *J* = 15 Hz, ^1^H), 7.90 (d, *J* = 15 Hz, ^1^H), 7.83 (dd, *J* = 10, 5 Hz, ^1^H), 7.72–7.66 (m, 2H), 7.63 (dd, *J* = 10, 5 Hz, ^1^H), 7.41 (d, *J* = 10 Hz, ^1^H), 7.39–7.34 (m, 2H), 7.28–7.22 (m, ^1^H).

**(*E*)-3-[3-(3-bromophenyl)acryloyl**]**-2H-chromen-2-one (3s).** Yellow solid; yield 41.8%;^1^H NMR (500 MHz, Chloroform-*d*) *δ* 8.61 (s, ^1^H), 7.94 (d, *J* = 15 Hz, ^1^H), 7.82–7.75 (m, 2H), 7.70–7.65 (m, 2H), 7.59 (d, *J* = 10 Hz, ^1^H), 7.53 (ddd, *J* = 3, 1.5, 1 Hz, ^1^H), 7.43–7.35 (m, 2H), 7.29 (t, *J* = 10, 10 Hz, ^1^H).

**(*E*)-3-[3-(4-bromophenyl)acryloyl**]**-2H-chromen-2-one (3t).** Yellow solid; yield 44.5%; ^1^H NMR (500 MHz, Chloroform-*d*) *δ* 8.61 (s, ^1^H), 7.95 (d, *J* = 15 Hz, ^1^H), 7.79 (d, *J* = 15 Hz, ^1^H), 7.70–7.65 (m, 2H), 7.54 (s, 4H), 7.41 (d, *J* = 10 Hz, ^1^H), 7.36 (td, *J* = 5, 1 Hz, ^1^H).

**(*E*)-3-[3-(thiophen-2-yl)acryloyl**]**-2H-chromen-2-one (3u).** Yellow solid; yield 40.7%; ^1^H NMR (500 MHz, DMSO-*d*_6_) *δ* 12.00 (s, ^1^H), 8.68 (s, ^1^H), 8.01 (d, *J* = 15 Hz, ^1^H), 8.10–8.04 (m, 2H), 7.98 (td, *J* = 10, 3.5 Hz, 2H), 7.78–7.73 (m, ^1^H), 7.69 (d, *J* = 15 Hz, ^1^H), 7.54–7.49 (m, 2H), 7.44 (td, *J* = 10, 1.5 Hz, ^1^H), 7.30–7.22 (m, 2H).

**(*E*)-3-[3-(** ^**1**^ **H-indol-2-yl)acryloyl**]**-2H-chromen-2-one (3v).** Yellow solid; yield 51.7%; ^1^H NMR (500 MHz, Chloroform-*d*) *δ* 8.60 (s, ^1^H), 7.92 (dd, *J* = 40, 20 Hz, 2H), 7.71–7.65 (m, 4H), 7.44–7.39 (m, 4H), 7.35 (t, *J* = 8, 7.5 Hz, ^1^H).

### α-Glucosidase Inhibition and Kinetics Mechanism Analysis Assay

The α-glucosidase inhibitory activity assay of coumarin-chalcone derivatives 3 (a∼v) was conducted using *p*-NPG as a substrate. (Pogaku et al., 2019; Saeedi et al., 2019; Xu et al., 2019). 10 μl of the test compound and 10 μl of the enzyme (final concentration 0.1 U/ml) were added to 130 μl of PBS (0.1 M phosphate, pH 6.8), and incubated at 37°C for 10 min. Then *p*-NPG (final concentration 0.25 mM) was added and the absorbance change was measured by a micro-plate reader at 405 nm. All experiments were assayed four times. The percentage of inhibition was obtained using the formula: Inhibition (%) = [(OD_1_ - OD_0_)/OD_0_] × 100%, where OD_1_ and OD_0_ represent the absorbance value of the experimental group and the blank group respectively. Acarbose as a positive sample was also tested. The IC_50_ value of each compound was obtained from the fitting curve of inhibition vs. compound concentration.

The type of inhibition was identified by the plots of enzymatic reaction rate (V) vs. α-glucosidase concentration. The test method was similar to the above enzyme activity assay. In the presence of different concentrations of compounds **3j**, **3q** and **3t**, respectively (0, 25, 30, and 40 μM), the absorbance change was detected under different concentrations of α-glucosidase (0.075, 0.1, 0.125, and 0.15 U/mL).

The inhibition mode was also detected using a similar method to that described above. In the presence of different concentrations of compounds **3j**, **3q** and **3t**, respectively (0, 25, 30 and 40 μM), the absorbance change was measured under different concentrations of *p*-NPG (0.25, 0.5, 0.75, 1 mM). The inhibition mode of the inhibitor was obtianed using Lineweaver-Burk plots. The constant K_I_ was obtained by secondary plots of the derivatives concentration (I) vs. Slope, constant K_IS_ was obtained by secondary plots of the derivatives concentration (I) vs. Intercept.

### Molecular Docking

The molecular docking of α-glucosidase with compounds **3d**, **3f**, and **3i** was simulated with Sybyl 2.1.1 (United States) and Pymol software. First, compounds **3d**, **3f**, and **3i** were prepared by energy minimization with the Tripos force field by the Powell gradient algorithm with Gasteiger-Hückel charges. The maximum iterations for the minimization were set to 10 000. The minimization was terminated when an energy gradient convergence criterion of 0.005 kcal mol−1 Å−1 was reached. The energy convergence criterion of 0.01 kcal/mol and the maximum iterations for the minimization of 1,000 times were reached. Next, the crystal structure of *Saccharomyces cerevisiae* isomaltase (PDB: 3AJ7) was downloaded from the RCSB Protein Data Bank. The protein was prepared by biopolymer and implemented following the procedure of removing water molecules, adding hydrogen atoms, and repairing end residues. The active pocket of protein was generated with the automatic mode. Then the molecular docking of protein with **3d**, **3f**, and **3i** was operated in the default format. The Pymol software was used to draw the view of protein with **3d**, **3f**, and **3i**.

## Data Availability

The original contributions presented in the study are included in the article/Supplementary Material, further inquiries can be directed to the corresponding authors.
