# Supplementary material for: Synthesis and Evaluation of Coumarin-Chalcone Derivatives as α-Glucosidase Inhibitors
Source: Front Chem. 2022 Jun 27;10:926543. doi: 10.3389/fchem.2022.926543 (PMC9271751; doi:10.3389/fchem.2022.926543)
Supplement: Supplementary file 1 [file DataSheet1.docx]

Supplementary Material

Synthesis and evaluation of coumarin-chalcone derivatives as α-glucosidase inhibitors

**Chun-Mei Hu †, Yong-Xin Luo †, Wen-Jing Wang，Jian-Ping Li, Meng-Yue Li , Yu-Fei Zhang , Di Xiao, Li Lu , Zhuang Xiong *, Na Feng*, Chen Li***

School of Biotechnology and Health Sciences, Wuyi University, Jiangmen 529000, P. R. China

†**Chun-Mei Hu and Yong-Xin Luo contributed equally to this work.**

*** Correspondence:**Corresponding Author: Zhuang Xiong, Na Feng, Chen Li.

E-mail: wyuchemxz@126.com; Tel./fax: +86 750 3299397.

1. **^1^H NMR of compouds………………………………………………………………………..2-12**

**2. Inhibitory mechanism analysis of 3j and 3q………………………………………………13-14**

**Fig.S1** 3a **(**^1^H NMR)

**Fig.S2** 3b **(**^13^C NMR)

**Fig.S3** 3c **(**^1^H NMR)

Fig.S4 3d (^1^H NMR)

**Fig.S5** 3e **(**^1^H NMR)

Fig.S6 3f (^1^H NMR)

**Fig.S7** 3g **(**^1^H NMR)

**Fig.S8** 3h **(**^1^H NMR)

**Fig.S9** 3i **(**^1^H NMR)

**Fig.S10** 3j **(**^1^H NMR)

**Fig.S11** 3k **(**^1^H NMR)

**Fig.S12** 3l **(**^1^H NMR)

**Fig.S13** 3m **(**^1^H NMR)

**Fig.S14** 3n **(**^1^H NMR)

**Fig.S15** 3o **(**^1^H NMR)

**Fig.S16** 3p **(**^1^H NMR)

**Fig.S17** 3q **(**^1^H NMR)

**Fig.S18** 3r **(**^1^H NMR)

**Fig.S19** 3s **(**^1^H NMR)

**Fig.S20** 3t **(**^1^H NMR)

**Fig.S21** 3u **(**^1^H NMR)

**Fig.S22** 3v **(**^1^H NMR)

**Fig.S23** Inhibition mechanism determination of compounds **3j** on α-glucosidase

**Fig.S24** Inhibition mechanism determination of compound **3q** on α-glucosidase

**Fig.S25** Lineweaver-Burk plots of compounds **3j** and **3q** on α-glucosidase (a). Plot of slope vs the concentration of compounds for the calculation of the inhibition constant *K*_I_ (b). Plot of intercept vs the concentration of compounds for the determination of the inhibition constant *K*_IS_ (c).
